# 1646. Optimizing Antibiotic Durations of Therapy in Pediatric Community-acquired Pneumonia

**DOI:** 10.1093/ofid/ofad500.1480

**Published:** 2023-11-27

**Authors:** Gregory Cook, Shubho Sarkar

**Affiliations:** Children's Hospital New Orleans, New Orleans, Louisiana; LSUHSC-NO, Children's Hospital New Orleans, New Orleans, Louisiana

## Abstract

**Background:**

Several recent high-quality studies have shown similar efficacy for a shorter, 5-day duration of antibiotics instead of the prior standard, 10 days, in pediatric community-acquired pneumonia (CAP). Despite high-quality literature support, implementing short courses into routine clinical practice remains challenging. This study aimed to assess the impact on the duration of antibiotics for CAP of a pilot clinical pharmacist discharge antibiotic prescription review and departmental education supporting short-course antibiotics for CAP.

**Methods:**

This was a single-center retrospective cohort study for patients admitted with an order for any antimicrobial for CAP. Patients were excluded if they were admitted to the pediatric intensive care unit or transferred during their course of care. The pre-intervention period was from June 2021 to June 2022, and the postintervention group was from August to October 2022. A one-time education session was hosted with the Pediatric Hospital Medicine department summarizing the literature supporting short courses. Statistical analysis included ANOVA, Fisher’s Exact, and Mann-Whitney U test as appropriate.

**Results:**

One hundred twenty-four patients were included, 85 in the pre-intervention and 39 in the post-intervention groups. The median age was two years old in both groups (p=0.2). No differences were present in the median length of stay, penicillin-allergy status, or vaccination status. Patients had a positive viral test 56% and 59%, respectively (p=0.8). Discharge attendings did differ and impact the total length of therapy (p< 0.001). Overall median antibiotic duration for CAP decreased from 8 to 5 days (p< 0.01). Inpatient median durations remained the same at 2 days (p=0.5), while outpatient durations fell from 6 to 4 days (p< 0.01). Patients that received over seven days for CAP decreased from 62% to 10% (p< 0.01). Treatment failure at 30 days occurred in 2 and 8% (p=0.16), respectively.

**Conclusion:**

A pilot discharge prescription review by clinical pharmacists and education supporting short courses for CAP effectively reduced the duration of antibiotic therapy. Consideration should be given to discharge antibiotic prescription reviews when adopting short courses for common pediatric infections.

**Disclosures:**

**Shubho Sarkar, MD**, Abbott Laboratories: Stocks/Bonds|Abbvie INC: Stocks/Bonds|Bristol-Myers Squibb: Stocks/Bonds|CVS Health Corp: Stocks/Bonds|Johnson & Johnson: Stocks/Bonds

